# Social support and depressive symptoms among family caregivers of older people with disabilities in four provinces of urban China: the mediating role of caregiver burden

**DOI:** 10.1186/s12877-019-1403-9

**Published:** 2020-01-02

**Authors:** Yaqin Zhong, Jian Wang, Stephen Nicholas

**Affiliations:** 1 School of Public Health, Nantong University, 9 Seyuan Road, Nantong, 210029 Jiangsu China; 2 Dong Furen Institute of Economic and Social Development, Wuhan University, 54 Dongsi Lishi Hutong, Beijing, 100010 China; 3 School of Economics and School of Management, Tianjin Normal University, West Bin Shui Avenue, Tianjin, 300074 People’s Republic of China; 4 Research Institute for International Strategies, Guangdong University of Foreign Studies, Baiyun Avenue North, Guangzhou, 510420 People’s Republic of China; 5 TOP Education Institute 1 Central Avenue Australian Technology Park, Eveleigh Sydney, NSW 2015 Australia; 6 Newcastle Business School, University of Newcastle, University Drive, Newcastle, NSW 2308 Australia

**Keywords:** Social support, Caregiver burden, Depressive symptoms

## Abstract

**Background:**

To examine the relationship between social support and depressive symptoms of Chinese family caregivers of older people with disabilities, and to evaluate the role of caregiver burden as a potential mediator of that relationship.

**Methods:**

A survey questionnaire was completed face-to-face by 567 primary family caregivers of older people with disabilities in four provinces in China. Covariates that may affect depressive symptoms, such as the characteristics of disabled people (socio-economic factors, functional and cognitive capacity) and caregivers (caregiver duration and self-rated health of caregivers) were collected. Social support was measured by the Multidimensional Scale of Perceived Social Support (MSPSS); depressive symptoms were assessed by the shortened 10 item version of Center for Epidemiological Studies Depression scale (CES-D); and the caregiver burden was assessed by the Zarit Burden Interview (ZBI).

**Results:**

The prevalence of depressive symptoms among caregivers was 37.7%. Higher levels of social support was negatively associated with lower depressive symptoms. This relationship was partially mediated by the caregiver burden, where higher levels of the caregiver burden were negatively associated with depressive symptoms. Furthermore, caregivers who were women, spent extended time in caregiving and were in poor health, reported significantly higher depressive symptoms.

**Conclusions:**

Our results indicated that social support was negatively associated with depressive symptoms in family caregivers and in the caregiver burden. The caregiver burden partially mediated the social support-depressive symptoms association. Interventions for family caregivers should include increasing social support, health monitoring and structured interventions to reduce the caregiver burden and attenuate family caregivers’ depressive symptoms.

## Background

The global phenomenon of an aging population has the dual effect of increasing the number of older people with physical and mental disabilities requiring care and increasing the number of caregivers to older people with disabilities. China’s population aged rapidly between 2000 and 2017, with life expectancy at birth increasing from 71.4 years to 76.7 years and the proportion of the population over 60 rising from 7.0 to 17.3% [1]. The number of older people with physical disabilities or serious cognitive impairments also increased quickly. In China, formal long-term care facilities are in short supply and families are the main sources for caregiving. Care of family members with disabilities at home is very common and is a reflection of Chinese culture, especially represented by familism and filial piety [2, 3]. There are positive aspects to caregiving at home, including bringing family members closer together and confirming the cultural expectations of respecting and caring for family members in Chinese society [4]. But providing care has been described as a stressful experience, which may erode the physical and psychological health of caregivers. The overall impact of these experiences has been termed the caregiver burden [5]. Like other groups prone to depression, such as individuals with other mental disorders, alcohol and substance users, those suffering chronic illnesses or abuse or traumatic events, and those with a genetic predisposition to depression, family caregivers to older family members with disabilities form a unique sub-group “at risk” of depression [6].

There is growing evidence that the caregiver burden imposes both physical and psychological costs on caregivers [7]. Depression is one of the most significant problems facing caregivers. Three papers using meta-analysis to examine the nationwide prevalence of depression among caregivers found the prevalence of depression was between 26 and 57% [8–10]. In China, one study conducted in Shenyang City found that 67.3% of caregivers of patients with cancer reported depressive symptoms [11]. In societies where family members are the primary caregivers to older family members with a disability, understanding the risk factors and coping strategies to attenuate depression for this critically important caregiver group is an important health and social priority.

There is a substantial literature on social support and depression [12, 13]. However, most social support—depression studies do not directly assess mediation influences. Studies found that as the caregiver burden increased, caregivers were more likely to suffer from depression [14, 15]. There is also evidence that higher social support predicted a lower caregiver burden [16–18], suggesting that social support can provide the basis for an effective intervention to reduce the caregiver burden [17]. It follows that caregiver burden is a potential mediator between social support and depressive symptoms. Based on surveys completed by 567 primary caregivers of older people with disabilities, our study uses a mediated model approach to examine the relationships between social support, the caregiver burden and depression in China. We assess whether social support lowers depressive symptoms and the caregiver burden, and whether those caregivers with a lower caregiver burden experience lower levels of depression [19]. We also consider other characteristics of people with disabilities being cared for, such as age, gender and cognitive function, and of the caregivers’ characteristics, including age, gender, the relation to older people with disabilities and caregiver duration, social support, the caregiver burden and depression in caregivers. We hypothesize that patient and caregiver factors can lead caregivers to support and psychological treatment resources, allowing caregivers to more effectively manage the stresses and burdens associated with their caregiving and to prolong their ability to care for disabled relatives by reducing the caregiver burden.

Previous studies in China have focused on caregiving for those with specific diseases, such as Alzheimer’s disease and stroke [20, 21], or focused on specific institutions, such as a department of ophthalmology [12]. Our study investigates whether social support for family caregivers of older people with all types of disabilities, reduces the caregiver burden and attenuates depression in caregivers.

### Social support and depression

Social support is the interpersonal resources accessed and mobilized when individuals attempt to deal with the everyday stresses and strains of life [12]. Social support can be conveyed through verbal and nonverbal communication, and through perceived or actual exchanges of physical or psychosocial resources, including information and knowledge [22]. Commonly, social support is provided by networks of family, friends, neighbors and community members [23]. In China, most disabled old people are cared for at home by family members or relatives. In contrast to western value systems with a focus on independence, the cultural norms of social obligation, reciprocity, loyalty and duty in China explain the substantial levels of family caregiving. Leaving older family members with disabilities home alone or in medical facilities is frowned upon in Chinese culture [24]. Coupled with underdeveloped community-based care facilities, Chinese families bear the major burden of caregiving [25, 26]. Previous studies show that increased social support was associated with lower depression and higher life satisfaction [27–29]. People with more social support are more likely to recover from stressful conditions [22]. From the above discussion, the following hypothesis follows:

- Hypothesis 1: Social support is negatively associated with depressive symptoms.

### Social support and caregiver burden

The overall impact of physical, psychological, and social demands on caregivers’ quality of life has been termed the caregiver burden, including the persistent stress, hardship, and negative experiences from providing care [30]. In general, predictors of the caregiver burden can be divided into two groups: care receivers’ variables, such as functional and cognitive impairment; and caregivers’ variables, including caregivers’ characteristics, health status, relationship with disabled people and social support. Caregiver social support is a key intervention target to reduce the caregiver burden. Previous studies have revealed that emotional, instrumental, formal and informal support were negatively associated with the caregiver burden [31–33]. Caregivers with less support were reported to have a significantly higher caregiver burden than those with stronger social support [19].

### Caregiver burden as a potential mediator between social support and depression

It has also been well documented that caregiver experiences are often associated with depressive symptoms. Previous studies [26, 34] have demonstrated a common core model framework in caregiving stress models, where the caregiver burden mediated the effects of stress on caregiving outcomes, including depression. Caregivers who are unable to use or adapt strategies to meet caring demands, face an increased caregiver burden that may affect depressive symptoms [7]. Although some research has been conducted in China on sub-groups of carers, such as for Alzheimer patients [12, 20, 21], the interconnections between social support, caregiver burden and depressive symptoms have not been well established for caregivers in general. For caregivers of family members with a disability, our study examines the caregiver burden as a mediator on the relationship between social support and caregiver depressive symptoms, after controlling for patient personal demographic and caregiver characteristics.

Specifically, we hypothesize:

- Hypothesis 2: The caregiver burden is positively associated with depressive symptoms.
- Hypothesis 3: The caregiver burden mediates the negative association between social support and depressive symptoms.

**Fig. 1:**
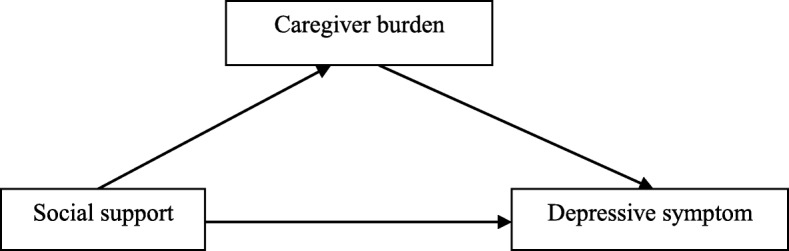
The theoretical model between social support, caregiver burden and depressive symptoms

Figure 1 sets out our model diagrammatically, which assumes that social support is associated with depressive symptoms and caregiver burden mediates the effects of social support on depressive symptoms.

## Methods

### Sampling and data collection

Between July and August, 2017, a cross-sectional study of caregivers was conducted in four Chinese provinces, Jiangsu, Anhui, Guizhou and Xinjiang. Jiangsu province is located in the east of China, representing the economically advanced coastal area, while Anhui province represents the central region with moderate levels of economic development. Provinces with significant ethnic minorities, Guizhou and Xinjiang, are located in western China, representing economically undeveloped regions. These provinces were chosen to represent China’s different economic and geographic characteristics, but may not be representative of all of China. Given the labor intensive costs of surveying whole provinces, in each province two cities were randomly selected and in each city about 80 disabled people 60 years and over and their caregivers were randomly identified. Supported by local community health service centers, the medical records of older people were reviewed to randomly draw a sample of people over 60 with a disability. People with disabilities were defined as those who were dependent for at least one activity of daily living, including dressing, bathing, eating, getting in and out of bed, moving inside the house, and toileting, and primary caregivers were their family members who took the main responsibility for taking care of the relative with the disability. Family members who were paid caregivers were excluded.

Face-to-face structured interviews were conducted in the caregivers’ home by trained interviewers from Nantong University. If the respondents could not read or write, the trained interviewers introduced the questions and helped them complete the answers. About 640 interviews were conducted, yielding 567 valid questionnaires from Jiangsu (150), Anhui (140), Guizhou (135) and Xinjiang (142), with an 88.6% response rate. The questionnaires include individual characteristics, activities of daily life (ADL), cognitive function of older people with disabilities, social support, caregiver burden, caregiving duration and health status of caregivers.

### Measurements

#### Depressive symptoms

The dependent variable, depressive symptoms, was assessed by the short version of the Center for Epidemiological Studies depression scale (CES-D) [35]. The short version of the CES-D includes 10 items about the symptoms of depression that occurred in the week before interview. Every item is rated on a 4-point scale (0 = rarely or less than 1 day; 4 = most of the time or 5–7 days). Two positive affect items were reverse-scored. Items were summed to give an overall score, where higher scores indicated higher levels of psychological distress and coded as a continuous variable.

#### Social support

The Multidimensional Scale of Perceived Social Support (MSPSS), which was developed by Zimet et al. (1988) [36], was used. The MSPSS consists of 12 questions to assess aspects of perceived social support, including support from family, friends and significant others [36]. The respondents were asked to rate each item on a 7-point Likert type scale ranging from 1 (very strongly disagree) to 7 (very strongly agree). The total score ranged from 12 to 84, with higher scores indicating more support. We used the Chinese version of MSPSS [37] and the reliability Cronbach alpha was 0.936.

#### Caregiver burden

Zarit et al. (1980) put forward an operational definition of caregiver burden and developed the Zarit Burden Interview (ZBI) to assess the caregiver burden [38]. The ZBI is one of the most widely used instruments for assessing the burden experienced by caregivers who take care of community residing older people with disabilities. The Chinese version of ZBI has been validated and found a practical instrument [39], with a Cronbach’s α of 0.903 in this study. The ZBI includes 22 questions about the impact of the old people’s disabilities on caregivers’ life. Caregivers reported each item on a five-point scale, ranging from 0 to 4 (0 = never, 1 = rarely, 2 = sometimes, 3 = quite frequently and 4 = nearly always). Total scores range from 0 to 88, with higher scores indicating increased caregiver burden. The degree of caregiver burden was divided into four categories: 0 to 20 (little or no burden), 21 to 40 (mild to moderate burden), 41 to 60 (moderate to severe burden), and 61 to 88 (severe burden) [38].

#### Other variables

To test our hypotheses, it was necessary to control for potential confounding factors that may affect the relationship between social support and depressive symptoms. The characteristics of older people with disabilities and caregivers were included as control variables, including personal characteristics, functional capacity and cognitive function of people with a disability and care hours per day, care duration, self-rated health of caregivers, because all of these factors have been shown to influence psychological well-being.

The Barthel Index (BI) was used to measure the functional capacity of people with a disability [40]. The BI comprises 10 items to measure independence for the activities of daily living (ADL). It has a range of 0–100, with higher scores indicate higher level of independence. The cognitive function of the people with a disability was measured by Pfeiffer’s Short Portable Mental Status Questionnaire (SPMSQ) [41]. The SPMSQ included 10 items, ranging from a score of 0 to 10. Individuals whose scores were less than three were considered ineligible for participation in the study.

### Ethical considerations

The study was approved by the Ethics Committee of Nantong University. All respondents were informed about the aim of the study and assured that the information would only be used for research purposes. Respondents were not identified and informed consent was obtained.

### Statistical analyses

Descriptive statistics and OLS regression were used to explore the association between social support, caregiver burden and depressive symptom in the study. The Karlson, Holm and Breen (KHB) [42, 43] method was used to assess the mediation role of caregiver burden. We centered the means of social support, caregiver burden and depressive symptoms, as well as Barthel index and cognitive function scores of older people with disabilities. In model 1, the dependent variable was depressive symptoms and the association between social support and depressive symptoms was explored. In model 2, the dependent variable was caregiver burden and the characteristics of older people with disabilities and caregivers were controlled. Model 3 further investigated the association between caregiver burden and depressive symptoms, while model 4 included all potential covariates. Personal and demographic variables were controlled in all regression models. Adjusted R-square was used to measure model fitness, which can be interpreted as the percent of variance in the response variable explained by the model. The dependent variables followed a normal distribution and tests for heterogeneity were negative.

For the mediation analyses, the KHB [42, 43] method was used to assess whether caregiver burden mediates the association between social support and depressive symptom. KHB provides unbiased decompositions of total effects into direct and indirect effects. By comparing the estimated coefficients obtained from the reduced model (without the mediator) to the full model (with the mediator), the decomposition is accomplished. An estimate of the indirect effect is the differences between these two sets of estimated coefficients. The proportion of mediating effect among the total effect was calculated as the indirect effect divided by the total effect. All analyses were conducted using Stata 14.0 at a 5% significance level.

## Results

### Characteristics of caregivers and disabled people

The personal and demographic characteristics of older people with disabilities and their primary caregivers are showed in Table 1. The average age of the disabled people was 80.6 years and there were more females (54.7%) than males (45.7%) in our sample. The average scores of the functional capacity BI was 23.2, which meant a high level of dependence, and the SPMSQ cognitive function was 6.2, or a moderate impaired cognitive function. Regarding caregivers, the average age was 62.6 and 59.3% were female. The average hours of care per day was 18.0. The care duration refers to the lifetime experience of a caregiver and the average care duration was 57.1 months. Thirty two percent of caregivers were spouses, 58% children and 10.1% other family members. Caregivers self-rated their health: 22.4% poor; 41.1% fair and 36.5% good.

**Table 1:** Characteristics of older people with disabilities and their primary caregivers (*n* = 567)

Variables	N (%)	Mean ± SD
Older people with disabilities		
Age		80.550 ± 8.778
Gender		
Male	259 (45.7)	
Female	308 (54.2)	
Barthel index		23.201 ± 30.693
Cognitive function		6.159 ± 4.157
Caregiver		
Age		62.614 ± 26.533
Gender		
Male	231 (40.7)	
Female	336 (59.3)	
Relation to care-recipients		
Spouse	181 (31.9)	
Children	329 (58.0)	
Others	57 (10.1)	
Care time per day (hours)		17.977 ± 7.888
Caregiver duration (months)		57.120 ± 73.227
Caregiver’s self-rated health		
Poor	127 (22.4)	
Fair	233 (41.1)	
Good	207 (36.5)	
Province		
Jiangsu	150(26.5)	
Anhui	140(24.7)	
Guizhou	135(23.8)	
Xinjiang	142(25.0)	

### Effects of social support and caregiver burden on depressive symptoms for caregivers

OLS regression analysis was conducted to examine the association between social support, caregiver burden and depressive symptoms. Model 1 in Table 2 included social support, depressive symptoms and characteristics of disabled people and caregivers. The negative coefficients suggest that social support was negatively associated with depressive symptoms. In model 2, the dependent variable was caregiver burden, where the negative coefficients indicate that more social support reduced the caregiver burden. In model 3, the results indicated that the caregiver burden gaves rise to higher levels of depressive symptoms. In model 4, all possible covariates were controlled and the results indicated that social support was negatively associated with depressive symptoms, while the caregiver burden was positively associated with depressive symptoms. Model 4 explained 50.89% of the variance on depressive symptoms. In regard to other variables, the functional capacity and cognitive function of disabled people were related to depressive symptoms. Caregivers who were female, spent extended time in caregiving and were in poor health, had more depressive symptoms. We found no significant differences across provinces.

**Table 2:** Association between social support, caregiver burden and depressive symptoms of caregivers

Variables	Model 1^a^		Model 2^b^		Model 3^a^		Model 4^a^	
	β	SE	β	SE	β	SE	β	SE
Social support	−0.399**	0.035	− 0.271**	0.037			−0.256**	0.031
Caregiver burden					0.609**	0.035	0.525**	0.034
Older people with disabilities								
Age	−0.012	0.042	−0.092*	0.043	0.048	0.037	0.036	0.035
Gender^1^: Female	−0.040	0.075	−0.060	0.079	−0.002	0.067	−0.009	0.063
Barthel index	0.020	0.044	−0.216**	0.046	0.138**	0.040	0.133**	0.038
Cognitive function	0.124**	0.041	0.025	0.043	0.122**	0.037	0.111**	0.035
Caregiver								
Age	0.020	0.039	−0.012	0.040	0.033	0.034	0.027	0.032
Gender^2^: Female	0.233**	0.075	0.124	0.078	0.162*	0.067	0.168**	0.063
Relation to disabled people^3^								
Children	0.040	0.101	0.340**	0.106	−0.190*	0.090	−0.139	0.086
Others	−0.122	0.142	0.118	0.148	−0.218	0.126	−0.184	0.119
Hours of care per day	−0.075	0.043	0.051	0.044	−0.134**	0.038	−0.101**	0.036
Caregiver duration	−0.053	0.036	0.061	0.038	−0.098**	0.032	−0.085**	0.031
Self-rated health^4^								
Fair	−0.327**	0.096	−0.140	0.100	−0.237**	0.085	−0.253**	0.081
Good	−0.892**	0.102	−0.676**	0.106	−0.460**	0.093	−0.537**	0.089
Adj R^2^	29.97%		23.65%		44.95%		50.89%	

1 and 2: reference = male; 3: reference = spouse; 4: reference = poor;

a: Dependent variable = depressive symptoms;

b: Dependent variable = caregiver burden;

*p < 0.05 **p < 0.01

Table 3 presents the results stratified by the different relationship to older people with disabilities. Model 1–3 show results for spouse, children and others family members. For spouse and children, social support was negatively associated with depressive symptoms. But this association was not significant for significant others. For all caregivers, the caregiver burden was positively associated with depressive symptoms.

**Table 3:** Association between social support, caregiver burden and depressive symptoms of caregivers (stratified by the different relation to care-recipients)

Variables	Model 1 (spouse)		Model 2 (children)		Model 3 (others)	
	β	SE	β	SE	β	SE
Social support	−0.283**	0.053	−0.246**	0.042	−0.111	0.122
Caregiver burden	0.556**	0.071	0.551**	0.044	0.398**	0.099
Older people with disabilities						
Age	0.066	0.068	0.001	0.057	0.027	0.094
Gender^a^: Female	−0.111	0.304	−0.065	0.083	0.192	0.232
Barthel index	0.141*	0.065	0.113*	0.052	0.176	0.128
Cognitive function	0.215**	0.059	0.056	0.048	0.046	0.111
Caregiver						
Age	0.015	0.034	0.129	0.152	0.161	0.308
Gender^b^: Female	−0.008	0.313	0.226**	0.079	−0.111	0.310
Hours of care per day	−0.182*	0.078	−0.080	0.043	−0.105	0.155
Caregiver duration	−0.084	0.054	−0.092*	0.040	−0.076	0.193
Self-rated health^c^						
Fair	−0.256*	0.128	−0.256*	0.113	−0.269	0.412
Good	−0.246	0.160	−0.613**	0.121	−0.614	0.392
Adj R^b^	49.87%		52.68%		29.84%	

^a^ and ^b^: reference = male; ^c^: reference = poor;

*p < 0.05 **p < 0.01

### The mediation role of caregiver burden between social support and depressive symptoms

**Fig. 2:**
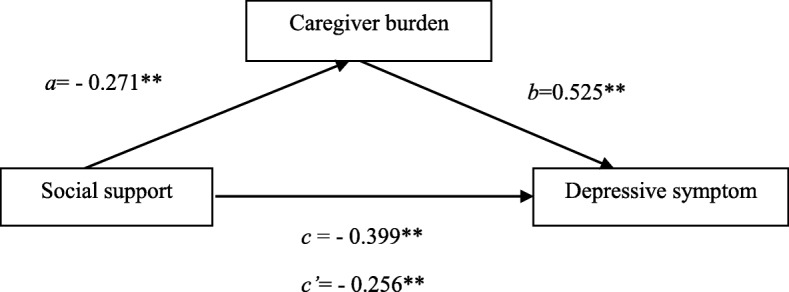
The mediating effects of caregiver burden on the relation between social support and depressive symptoms

Figure 2 shows diagrammatically the results of our theoretical model: path *a* shows the direct link between social support and caregiver burden; path *b* shows the link between caregiver burden and depressive symptom; and path *c* represented the link between social support and depressive symptom. *c’* represents the effect of social support on depressive symptoms including the mediating caregiver burden. When excluding the caregiver burden, depressive symptoms respond primarily to social support (path *c,* total effect), however, once caregiver burden is considered, the coefficient (path *c*) was reduced to *c’* (direct effect). Using the KHB [42, 43] method in Table 4, the caregiver burden partially mediated the association between social support and depressive symptoms. As shown in Table 4, the mediating effect (indirect effect, *ab* = *c* - *c’*) of the caregiver burden was 0.142, which accounted for 35.59% of the total effect.

**Table 4:** Models of the mediating role of caregiver burden in the relationship between social support and depressive symptoms

	β	SE	Z	*P*	OR (95%CI)
Total effect (*c*)^a^	−0.399	0.020	−13.41	< 0.001	0.671(0.633–0.712)
Direct effect (*c’*)^a^	−0.256	0.024	−8.23	< 0.001	0.774(0.728–0.823)
Indirect effect (*ab* = *c* –*c*’)^a^	− 0.142	0.867	0.018	< 0.001	0.867(0.832–0.904)

^a^See Fig. 2 and text for further explanation

## Discussion

Given the unique role of Chinese family members as caregivers of older family members with disabilities, our study analysed the association between social support and depressive symptoms for caregivers in China and determined whether the caregiver burden mediated this association. We are not aware of similar studies of China family caregivers’ mental state when looking after relatives with disabilities. Our results displayed the same inverse relationship between social support and depression found in non-Chinese studies [13, 22, 44, 45]. Consistent with these studies, we found that positive social support improved the mental health of caregivers of family members with disabilities [46]. Our results identified ‘at risk’ caregivers, such as females, those spending extended time in caregiving and those in poor health, who would benefit from enhanced social support. People with more social support are more likely to recover from stress and depression; those without adequate social support may suffer an exacerbation in their psychosocial distress and depression.

Both western studies [33, 47–49] and our findings strongly suggest that social support was significantly associated with a lower caregiver burden [38, 39]. Similar to other studies [38, 50], we found that social support from families, friends and others was beneficial for caregivers, providing access to resources, information and knowledge. High levels of social support have been reported to have a buffering effect on the caregiver burden [51], assisting caregivers to cope with challenges in the caregiving experience, including the impact on their physical health, psychological and emotional wellbeing, social isolationism and financial stress [52].

Also consistent with non-Chinese studies [7, 33, 53], the care recipients’ functional disability and cognitive function and the gender, caregiver duration and self-rated health of caregivers directly influenced caregivers’ depressive symptoms. When the care-recipient’s functional status and cognitive function were low, the caregiver’s burden increased. For example, as care recipients’ functional disability and cognitive impairment increased the number of care activities that the caregiver was required to perform rose, squeezing out time to adjust their other family, work and social obligations and experiencing a higher care burden. Caregivers’ health status was associated with higher level of caregiver burden, with those in poor health finding it difficult to complete caregiving tasks because of their own physical and psychological health limitations. Like other studies [54], our result show that depressive symptoms increased with the duration of caregiving and the poor self-rated health of caregivers. Based on our results, we suggest clinicians target specific interventions to improve various aspects of caregivers’ mental health. We recommend that caregivers should be tested for depressive symptoms; health interventions should be organized for caregivers in poor health; and structured programs of social support organized for caregivers.

When we took the type of family caregiver-older disabled persons relationship into consideration, we found an association with social support and caregiver burden for spouses and adult child caregivers. As found in other studies, our research confirms the key role of social support for spouses and adult child caregivers, which can lower their caregiver burden, improve their quality of life and mediate depressive symptoms [55]. In contrast to western countries, spouse and adult child caring has likely reached its limit in Chinese society, due to the existing high levels of family caregiving support, urban migration of adult children that has left parents behind in rural villages and increasing income levels that will weaken intra-family reciprocity. China should expand non-family support strategies for caring for older persons with disabilities, such as respite care or paid home care. Such alternative non-family support mechanisms will attenuate the family members’ caregiver burden, which directly and indirectly impacts the welfare and mental health of caregivers [56]. For other family members, there was an association between depression and caregiver burden, but not with social support. In contrast to spouses and adult children, we speculate that other family member caregivers might have had wider outside the household connections, providing enhanced access to resources and information, as well as displaying different grief and health characteristics and different time and type of contact with the care recipients [57]. Unlike spouses and adult child caregivers, other family caregivers may not have been the key decision-makers regarding the disabled person’s care, which reduced their need to access resources and information and their stress levels. While outside the family links reduced the need for social support, it did not reduce the actual burden of caring for a family member with a disability, where the caregiver burden might increase depressive symptoms.

Our study revealed that the caregiver burden had a mediating role on social support and depressive symptoms. Caregivers burdened by financial, social, physical, physiological and emotional needs require high levels of social support to both reduce the caregiver burden and to attenuate depressive symptoms [7, 55]. Inversely, family caregivers with a low caregiver burden, perhaps only suffering financial stress or adverse physical health, require few social support resources to address the caregiver burden, but high levels of social support to reduce depression [51, 58]. The mediated role of caregiver burden suggests different types of social support differentially impact the caregiver burden versus depression symptoms. For example, access to financial loans to address financial stress or male carers gaining part-time flexible work will address the caregiver burden, but different types of social support, such psychoeducational interventions, are required to address depression arising from self-efficacy among female spousal carers [59]. A better understanding the mediating role of the caregivers burden, will allow social support mechanisms to be shaped to either addressing the caregiver burden and/or depressive symptoms. Our results also suggest that types of social support to address depression and the caregiver burden is likely to be different for spouse or adult child caregivers and other family members. More research is required to reveal the factors determining social support best suited to alleviating the caregiver burden versus tackling depression for caregivers of older people with disabilities.

### Strengths and limitations

In China, caregiving to older family members wih depression differs from that in western countries due to family members as the principal caregivers. The strengths of the present study include the relatively large sample, the study of four Chinese provinces, the high response rate and the use of validated scales to measure social support, caregiver burden and depressive symptoms. Several limitations should be stated. First, because of the cross-sectional design, the effects in our study does not mean social support had a causal influence on depressive symptoms, but signals a social support—depression relationship. Second, we relied on a self-reported depressive symptom scale instead of a psychiatric assessment of depression. Third, other possible mechanisms through which the effects of social support may ultimately affect depressive symptoms were not explored in the current study. Lastly, the surveys were only conducted for urban communities and only for mainland China, so studies of rural caregivers and further provinces need to be added to ensure our outcomes are nationally representative.

## Conclusions

China is unique in that caregiving of older people with disabilities is mainly provided by family members. Social support was negatively associated with depressive symptoms in family caregivers of old family members with disabilities. The caregiver burden mediated this association. By increasing social support of caregivers, the caregiver burden would be attenuated and also the depressive symptoms of caregivers lessened. Health professionals should be aware that the lack of adequate formal and informal social support can be detrimental to caregivers, which can impact the family caregiver, the family members with disabilities and other members of the family. It is important that health professionals identify caregivers at risk of depression and structure a program of adequate formal social support. The type of formal social support and effective family-based interventions will likely differ across families and carers, requiring caregiver-specific health checks, tailored social support and whole-of-family programs to mediate the caregiver burden. In terms of social service and health policy in China, our study suggests the need to enhance formal support mechanisms, which will require investments in health care facilities, respite homes and community centers. There will also need to be a shift in community opinion, changing traditional views about the care of relatives with disabilities. Social policy will need to guide and promote a change in society’s moral views about care for family members with disabilities, which balances family care with increased government responsibilities for the care of people with disabilities.

## Data Availability

The data generated and analyzed in the current study are not publicly available. Please contact with corresponding author for the data.

## Abbreviations

ADL: activities of daily life
CES-D: Center for Epidemiological Studies Depression scale
MSPSS: Multidimensional Scale of Perceived Social Support
ZBI: Zarit Burden Interview
